# Longitudinal prediction of depressive symptoms in older age (80+) using psychological, biological and functional variables

**DOI:** 10.1002/alz70857_104479

**Published:** 2025-12-25

**Authors:** Philip Zeyen, Lena Sannemann, Xiaochen Hu, Michelle Gerards, Judith Wenner, Michael Wagner, Christiane Woopen, Susanne Zank, Frank Jessen, Forugh S Dafsari

**Affiliations:** ^1^ University of Cologne, Faculty of Medicine and University Hospital Cologne, Department of Psychiatry, Cologne, Germany; ^2^ University of Cologne, Faculty of Medicine and University Hospital Cologne, Cologne, Germany; ^3^ Department of Psychiatry and Psychotherapy, Medical Faculty, University of Cologne, Cologne, Germany; ^4^ ceres ‐ Cologne Center for Ethics, Rights, Economics and Social Sciences of Health, University of Cologne, Cologne, NRW, Germany; ^5^ Institute of Sociology and Social Psychology, University of Cologne, Cologne, Germany, Cologne, NRW, Germany; ^6^ Center for Life Ethics, University of Bonn, Bonn, Germany; ^7^ Department of Special Education and Rehabilitation Science, Faculty of Human Sciences, University of Cologne, Cologne, Germany, Cologne, NRW, Germany; ^8^ Department of Psychiatry, Medical Faculty, University of Cologne, Cologne, Germany; ^9^ Department of Psychiatry, University of Cologne, Medical Faculty, Cologne, Germany

## Abstract

**Background:**

Late‐life depression (LLD) is a common disorder, especially in the age group of 80 years and older. The aim of this analysis is the longitudinal evaluation of predictors of LLD in a population‐representative sample.

**Methods:**

In the NRW80+ study, participants (age≥80 years) representative for the German state of North Rhine‐Westphalia were comprehensively characterised at two time points (baseline: 2017 (*n* = 1863); follow‐up/FU: 2019/2020 (*n* = 912)). Recruitment was based on 55 randomly selected community registries. The interviews were conducted by computer‐assistance. We analysed data from 680 participants who were either cognitively unimpaired (CU) according to the DemTect test, had mild cognitive impairment (MCI) or dementia and had depression scores available at baseline as well as FU. Depression symptoms were assessed by the ‘Depression in Aging Scale’ (DIA‐S).

In previous analyses, 21 variables collected at baseline were identified in a data‐driven procedure and divided into four different areas (function, values/lifestyle, autonomy/satisfaction and biological/somatic variables). To predict depressive symptoms at FU (DIA‐S score ≥2/4), binary regression analyses were carried out for each diagnostic group with the four areas at baseline as predictors.

**Results:**

Among the 680 participants, 79.0% were CU, 13.2% had MCI and 7.4% had dementia. There were significant group differences for the presence of depressive symptoms at FU between the different diagnostic groups (CU: 19.6%, MCI: 31.2%, dementia: 30.0%; *p* = 0.016).

The binary regression analyses for the CU group indicated that the psychological variables (autonomy/satisfaction) explained the highest variance for depressive symptoms at FU (Nagelkerke's *R* ^2^ = 0.149). Contrary, in the MCI group, the biological‐somatic variables explained 29.7% (Nagelkerke's *R^2^* = 0.297) and values/lifestyle variables 32.1% of the variance at FU (Nagelkerke's *R^2^* = 0.321), which was significantly higher than the variance explained by the autonomy/satisfaction model (Nagelkerke's *R^2^* = 0.224). The dementia diagnosis group did not show any significant regression models.

**Conclusion:**

For the longitudinal prediction of depressive symptoms, psychological variables contribute most significantly in the CU group, whereas in the MCI group, biological‐somatic and values/lifestyle variables are more relevant. This could lead to new perspectives in prevention of LLD.

